# Post‐stem cell transplant maintenance in FLT3^mut^ acute myeloid leukemia – A retrospective analysis: Outcomes are improved with midostaurin but not with gilteritinib

**DOI:** 10.1002/jha2.885

**Published:** 2024-04-08

**Authors:** Karam Ashouri, Krithika Chennapan, Anastasia Martynova, Samvel Nazaretyan, Amir Ali, Anush Aram Ginosyan, Eric Tam, Abdullah Ladha, Karrune Woan, Preet Chaudhary, Imran Siddiqi, George Yaghmour

**Affiliations:** ^1^ University of Southern California Keck School of Medicine Los Angeles California USA; ^2^ Internal Medicine Jane Anne Nohl Division of Hematology and Center for the Study of Blood Diseases University of Southern California Norris Comprehensive Cancer Center Los Angeles California USA; ^3^ Department of Pathology University of Southern California Keck School of Medicine Los Angeles California USA

**Keywords:** AML, FMS‐like tyrosine kinase 3 (FLT3) inhibitor, gilteritinib, HSCT, maintenance, midostaurin

## 1 BACKGROUND

The FMS‐like tyrosine kinase 3 (*FLT3*) domain is the most mutated gene in acute myeloid leukemia (AML), with FLT3 internal tandem duplication (ITD) mutations conferring adverse outcomes [1, 2]. Maintenance therapy after hematopoietic stem cell transplant (HSCT) may be essential, as FLT3 AML patients experience high rates of post‐HSCT relapse and mortality [3]. Terao et al. found that relapsed/refractory (R/R) FLT3 AML patients who received post‐HSCT maintenance gilteritinib had improved overall survival (OS) (1‐year OS, 100% vs. 45.5%, *p* = 0.0075) and cumulative incidence of relapse (CIR, 1‐year CIR 0% vs. 68.8%, *p* = 0.0028) [4]. However, the phase 3 MORPHO trial failed to reach the primary outcome of improved relapse‐free survival (RFS) with post‐HSCT maintenance gilteritinib compared to placebo in FLT3‐ITD AML patients except in measurable residual disease (MRD) positive patients [5]. As prospective data on FLT3 inhibitor maintenance therapy are evolving, there is a need to investigate real‐life data and outcomes to guide clinical decisions. 

## 2 METHODS

We retrospectively studied adult patients with FLT3 AML treated at the University of Southern California (USC) Norris Cancer Center between May 2017 and July 2022. This study was approved by USC's Institutional Review Board, and data were retrieved from the Norris Comprehensive Cancer Center's electronic medical record system. 

## 3 RESULTS

### 3.1 Patient population

Of the 40 patients included in this study, 30 (75%) were FLT3‐ITD positive, and 10 (25%) were FLT3‐tyrosine kinase domain (FLT3‐TKD) positive. Most patients (67.5%) were in first complete remission (CR1) and 95% were MRD negative at the time of HSCT. All patients received a FLT3 inhibitor pre‐HSCT. Twenty‐nine patients received post‐HSCT maintenance therapy with an FLT3 inhibitor initiated at a median of D+104 (range 56–384) while eleven did not receive an inhibitor (Figure S1). Among maintenance patients, 18 (62%) received gilteritinib and 11 (38%) received midostaurin. Besides race (*p* = 0.022), there was no significant difference in baseline characteristics between patients who received FLT3 maintenance and those who did not (Table 1). The decision to withhold FLT3 inhibitor maintenance therapy from a subset of patients was made through physician clinical judgment, and the majority did not start the inhibitor due to post‐SCT cytopenias. Patients were followed for a median of 26.2 months. The median OS and RFS were not reached (NR), whereas the median graft versus host disease‐free, relapse‐free survival (GRFS) was 29.3 months (95% Confidence Interval [CI] 26.5%‐NR). The 1‐year GRFS, RFS, and OS were 69.6% (95% CI 56.6–85.6), 85.0% (95% CI 74.6–96.8), and 89.6% (95% CI 80.4–99.8), respectively.

**TABLE 1:** Demographics stratified by FMS‐like tyrosine kinase 3 (FLT3) maintenance status.

	No FLT3 inhibitor maintenance (*N* = 11)	FLT3 inhibitor maintenance (*N* = 29)	Total (*N* = 40)	*p*‐Value
**Age at SCT, years**	48.0 (33.0, 67.0)	46.0 (30.0, 69.0)	47.0 (30.0, 69.0)	0.832^a^
**Sex**				0.293^b^
Female	4 (36.4%)	17 (58.6%)	21 (52.5%)	
Male	7 (63.6%)	12 (41.4%)	19 (47.5%)	
**Race/Ethnicity**				0.022^b^
Asian	2 (18.2%)	0 (0.0%)	2 (5.0%)	
Black	0 (0.0%)	4 (13.8%)	4 (10.0%)	
Hispanic	8 (72.7%)	14 (48.3%)	22 (55.0%)	
White	1 (9.1%)	11 (37.9%)	12 (30.0%)	
**CR1/CR2 before SCT**				1.000^b^
CR1	7 (63.6%)	20 (69.0%)	27 (67.5%)	
CR2	4 (36.4%)	9 (31.0%)	13 (32.5%)	
**FLT3 mutation type**				1.000^b^
ITD	8 (72.7%)	22 (75.9%)	30 (75.0%)	
TKD	3 (27.3%)	7 (24.1%)	10 (25.0%)	
**NPM1 positive**				0.148^b^
No	9 (81.8%)	15 (51.7%)	24 (60.0%)	
Yes	2 (18.2%)	14 (48.3%)	16 (40.0%)	
**Number of pathogenic mutations (excluding FLT3)**	1.0 (0.0, 4.0)	2.0 (0.0, 5.0)	2.0 (0.0, 5.0)	0.657^a^
**ELN category 2022**				0.689^b^
Favorable	0 (0.0%)	3 (10.3%)	3 (7.5%)	
Intermediate	9 (81.8%)	22 (75.9%)	31 (77.5%)	
Adverse	2 (18.2%)	4 (13.8%)	6 (15.0%)	
**Donor type**				0.905^b^
Haplo SCT	3 (27.3%)	11 (37.9%)	14 (35.0%)	
MRD	4 (36.4%)	8 (27.6%)	12 (30.0%)	
MUD	4 (36.4%)	10 (34.5%)	14 (35.0%)	
**CNS disease**				0.178^b^
No	9 (81.8%)	28 (96.6%)	37 (92.5%)	
Yes	2 (18.2%)	1 (3.4%)	3 (7.5%)	
**Extramedullary disease**				0.686^b^
No	8 (72.7%)	23 (79.3%)	31 (77.5%)	
Yes	3 (27.3%)	6 (20.7%)	9 (22.5%)	
**Type of conditioning**				0.451^b^
MA	9 (81.8%)	19 (65.5%)	28 (70.0%)	
RIC	2 (18.2%)	10 (34.5%)	12 (30.0%)	
**Radiation type**				0.595^b^
FTBI	4 (36.4%)	6 (20.7%)	10 (25.0%)	
None	5 (45.5%)	14 (48.3%)	19 (47.5%)	
TBI	2 (18.2%)	9 (31.0%)	11 (27.5%)	
**Blast Percentage**	74.8 (25.0, 91.0)	70.0 (10.0, 95.0)	70.5 (10.0, 95.0)	0.844^a^
**FLT3 Inhibitor Type before SCT**				0.438^b^
Gilteritinib	0 (0.0%)	3 (10.3%)	3 (7.5%)	
Midostaurin	7 (63.6%)	20 (69.0%)	27 (67.5%)	
Gilteritinib and Midostaurin	2 (18.2%)	5 (17.2%)	7 (17.5%)	
Sorafenib	2 (18.2%)	1 (3.4%)	3 (7.5%)	
**MRD status at SCT**				0.452^b^
Negative	9 (90.0%)	28 (96.6%)	37 (94.9%)	
Positive	1 (10.0%)	1 (3.4%)	2 (5.1%)	
**Acute GVHD**				1.000^b^
No	4 (36.4%)	12 (41.4%)	16 (40.0%)	
Yes	7 (63.6%)	17 (58.6%)	24 (60.0%)	
**Chronic GVHD**				1.000^b^
No	9 (81.8%)	23 (79.3%)	32 (80.0%)	
Yes	2 (18.2%)	6 (20.7%)	8 (20.0%)	
**HCT Comorbidity Index**	0.0 (0.0, 5.0)	1.0 (0.0, 5.0)	1.0 (0.0, 5.0)	0.310^a^

Abbreviations: CNS, central nervous system; CR1, first complete remission; CR2, second complete remission; ELN, European LeukemiaNet; FLT3, FMS‐like tyrosine kinase 3; FTBI, fractionated total body irradiation; GVHD, Graft‐versus‐host disease.; Haplo, haploidentical; MA, myeloablative; MRD, measurable residual disease; MSD, match sibling donor; MSD, match unrelated donor; NPM, nucleophosmin; RIC, reduced intensity conditioning; SCT, stem cell transplant; TBI, total body irradiation.

^a^ Kruskal‐Wallis rank sum test.

^b^ Fisher's Exact Test.

### 3.2 FLT3 maintenance survival outcomes

**FIGURE 1:**
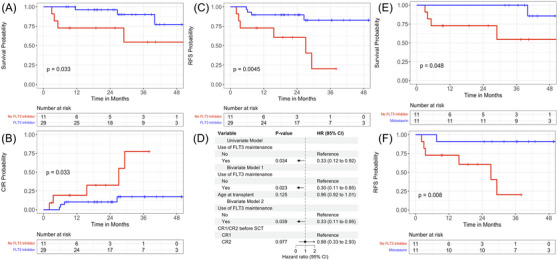
Clinical impact of FMS‐like tyrosine kinase 3 (FLT3) inhibitor maintenance therapy after hematopoietic stem cell transplant. (A) Overall survival (OS) from time of transplant in FLT3 inhibitor group versus no FLT3 inhibitor group (B) Cumulative incidence of relapse (CIR) from time of transplant in FLT3 inhibitor group versus no FLT3 inhibitor group (C) Relapse‐free survival (RFS) from time of transplant in FLT3 inhibitor group versus no FLT3 inhibitor group (D) Forrest plot evaluating graft versus host disease‐free, relapse‐free survival (GRFS) as predicted by FLT3 inhibitor maintenance use in addition to bivariate models controlling for age and stage pre‐transplant (E) OS from time of transplant in Midostaurin group versus no Midostaurin group (F) RFS from time of transplant in Midostaurin group versus no Midostaurin group.

Patients who received a FLT3 inhibitor as post‐HSCT maintenance had significantly improved OS (1‐year OS 96.2% [95% CI 89.0%–100%] vs. 72.7% [95% CI 50.6%–100%]; *p* = 0.033), CIR (*p* = 0.033), and RFS (1‐year RFS 89.7% [95% CI 79.2%–100%] vs.72.7% [95% CI 50.6%–100%]; *p* = 0.005), compared to those who did not receive maintenance (Figure 1A–C, respectively). Using Cox survival regression, FLT3 maintenance predicted GRFS (hazard ratio [HR] = 0.33, *p* = 0.034, Figure 1D – Univariate Model) and remained significant when controlling for age (HR = 0.30, *p* = 0.023, Figure 1D – Bivariate Model 1) and CR status pre‐transplant (HR = 0.33, *p* = 0.039, Figure 1D—Bivariate Model 2). 

### 3.3 Subgroup CR1 versus CR2 w/ and w/o maintenance

CR1 patients who received FLT3 maintenance demonstrated improved OS (100% [CI 100%–100%] vs 71.4% [CI 44.7%–100%]; *p* = 0.002, Figure S2A) and RFS (95% [CI 85.9%–100%] vs. 71.4% [CI 44.7%–100%]; *p* < 0.001, Figure S2B) versus those who did not. However, FLT3 maintenance did not improve OS or RFS in CR2 patients (*N* = 13) (*p* = 0.99, *p* = 0.8, Figure S3A,B).

### 3.4 Subgroup of midostaurin versus gilteritinib

Midostaurin showed improved OS and RFS compared to no FLT3 maintenance (*p* = 0.048, *p* = 0.008, Figure 1E,F). However, gilteritinib showed no OS or RFS benefit compared to no FLT3 maintenance (*p* = 0.22, *p* = 0.17, Figure S4A,B).

### 3.5 Comparison group Oregon Health and Science University

Given the group of patients without maintenance FLT3 inhibitors may be subject to selection bias, our FLT3 maintenance cohort was also compared to a 2018 public data cohort of patients from Oregon Health and Science University (OHSU) who did not receive FLT3 maintenance therapy because they were transplanted prior to the availability of maintenance therapy (Table S1). The cohorts had similar distributions in age, sex, ELN risk category, and type of FLT3 mutations, although the USC group had different types of HSCT (*p* = 0.003). Our maintenance patients had improved survival compared to the OHSU cohort (*p* = 0.016) (Figure S5).

### 3.6 FLT3 inhibitor discontinuation

Eight patients (three midostaurin, four gilteritinib, and one both) discontinued FLT3 inhibitors due to side effects, resulting in a shorter length of post‐HSCT FLT3 inhibitor use (116 days vs. 230 days, *p* = 0.005). The side effects leading to discontinuation were diverse, including gastrointestinal symptoms (*n* = 2), cytopenias (*n* = 2), rash (*n* = 1), pneumonitis (*n* = 1), confusion (*n* = 1), and neuropathy (*n* = 1).

## 4 DISCUSSION

We found post‐HSCT maintenance therapy with FLT3 inhibitors improved OS, with a 2‐year OS of 96.2% (93.3% for gilteritinib and 100% for midostaurin). The RADIUS trial reported a lower 2‐year OS of 85% with midostaurin maintenance, while SORMAIN reported 90.5% OS with sorafenib maintenance [6, 7]. Our 2‐year RFS with FLT3 maintenance was 89.7% (88.9% for gilteritinib and 90.9% for midostaurin), whereas both RADIUS and SORMAIN trials reported 2‐year RFS of 85%. The RADIUS study exclusively included patients with FLT3‐ITD mutations, which have poorer prognosis and may have contributed to worse outcomes than our study population (75% ITD and 25% TKD). However, our cohort included R/R AML patients and post‐HSCT maintenance with both midostaurin and gilteritinib, while the RADIUS population was exclusively in CR1 and used only post‐HSCT midostaurin. This is significant because neither our R/R patients nor our post‐HSCT gilteritinib patients had statistically significant improved survival. Therefore, survival benefit was exclusively derived from patients in CR1 and who received midostaurin.

Terao et al. found a survival benefit of gilteritinib maintenance in the post‐HSCT setting. The ADMIRAL trial also found a survival benefit for gilteritinib in R/R FLT3 AML, though less pronounced than Terao's, with only 40% achieving RFS [8, 9]. Contrastingly, our study found a survival benefit with post‐HSCT midostaurin maintenance but no benefit with gilteritinib. Possibly explaining this difference, post‐HSCT maintenance therapy initiation occurred later in our study, with a median of D+104 compared to D+55 in the ADMIRAL trial and D+36 in Terao's study. Terao proposes that early initiation of gilteritinib may augment the graft‐versus‐leukemia effect, which may yield improved survival outcomes in their study compared to others using post‐HSCT maintenance. However, this phenomenon has only been suggested in pre‐clinical studies so far [10]. Given the uncertainty regarding the optimal timing of FLT3 inhibitor maintenance therapy, delayed initiation of gilteritinib in our cohort possibly eliminated survival benefits. Our retrospective study demonstrates improved OS and RFS with post‐HSCT midostaurin maintenance therapy in FLT3‐positive AML. Further studies are required to elucidate which patients are likely to benefit from maintenance therapy with FLT3 inhibitors.

## 5 LIMITATIONS

This observational study with a limited sample of patients reflects single‐center practice. The treating clinician decided administration of maintenance FLT3 inhibitor therapy, the choice of inhibitor, and the dose and timing of initiation. Additionally, donor factors may have affected outcomes in the study population but were not evaluated in this study.

## AUTHOR CONTRIBUTIONS

All authors participated in study design, data acquisition, analysis, or interpretation of data, and contributed to manuscript writing and reviewing. All authors approved the final version of the manuscript.

## CONFLICT OF INTEREST STATEMENT

George Yaghmour Speakers Bureau: Jazz, Incyte, Astellas, BMS, Secura bio, blueprint, SOBI, Karius, Kite, Celgene, AbbVie, Rigel, Servier, GSK, Takeda, and Pfizer Advisory Board: Gilead, Alexion, Pfizer, AbbVie, and Servier

## FUNDING INFORMATION

Not applicable

## ETHICS STATEMENT

Not applicable

## PATIENT CONSENT STATEMENT

This study was IRB‐exempt from patient consent due to its retrospective nature.

## CLINICAL TRIAL REGISTRATION

The authors have confirmed clinical trial registration is not needed for this submission.

## Supporting information



Supporting Information

## Data Availability

Raw data were generated at USC. Derived data supporting the findings of this study are available from the corresponding author (George Yaghmour) upon request.
