# Correction to: Post‐stem cell transplant maintenance in FLT3^mut^ acute myeloid leukemia – A retrospective analysis: Outcomes are improved with midostaurin but not with gilteritinib

**DOI:** 10.1002/jha2.948

**Published:** 2024-05-31

Ashouri K, Chennapan K, Martynova A, Nazaretyan S, Ali A, Ginosyan AA, et al. “Post‐stem cell transplant maintenance in FLT3^mut^ acute myeloid leukemia – A retrospective analysis: Outcomes are improved with midostaurin but not with gilteritinib.” *Eur J Haematol*. 2024;5(2):423–27. https://doi.org/10.1002/jha2.885

The title of the letter should read “Post‐hematopoietic stem cell transplant FLT3 inhibitor maintenance therapy improves outcomes in acute myeloid leukemia: Real‐world insights and clinical implications.”

We apologize for this error.

