# Supplementary material for: Post‐stem cell transplant maintenance in FLT3mut acute myeloid leukemia – A retrospective analysis: Outcomes are improved with midostaurin but not with gilteritinib
Source: EJHaem. 2024 Apr 8;5(2):423–7. doi: 10.1002/jha2.885 (PMC11020142; doi:10.1002/jha2.885)
Supplement: Supplementary file 1 — Supporting Information [file JHA2-5-423-s001.docx]

Supplementary Tables/Figures:

**Figure S1. Flow chart of FLT3 inhibitor and reason for patients not receiving FLT3 maintenance** 
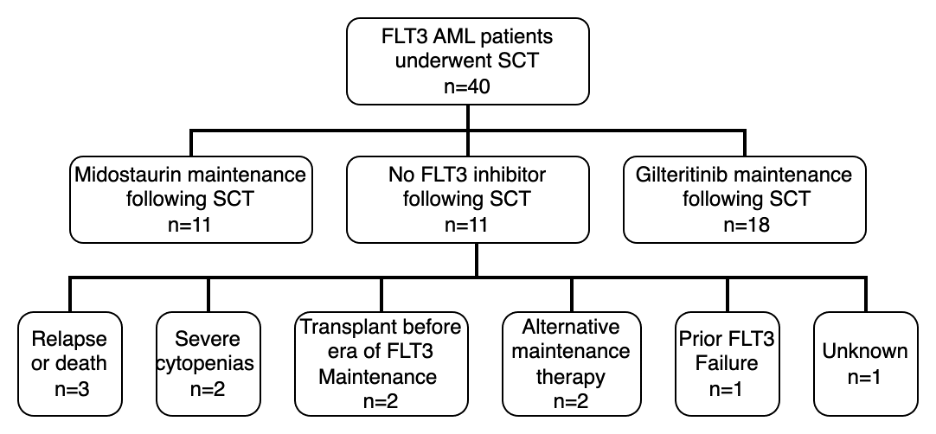


**Figure S2. Clinical impact of FLT3 inhibitor maintenance therapy after hematopoietic stem cell transplant in CR1 subgroup. (A) OS (B) RFS**


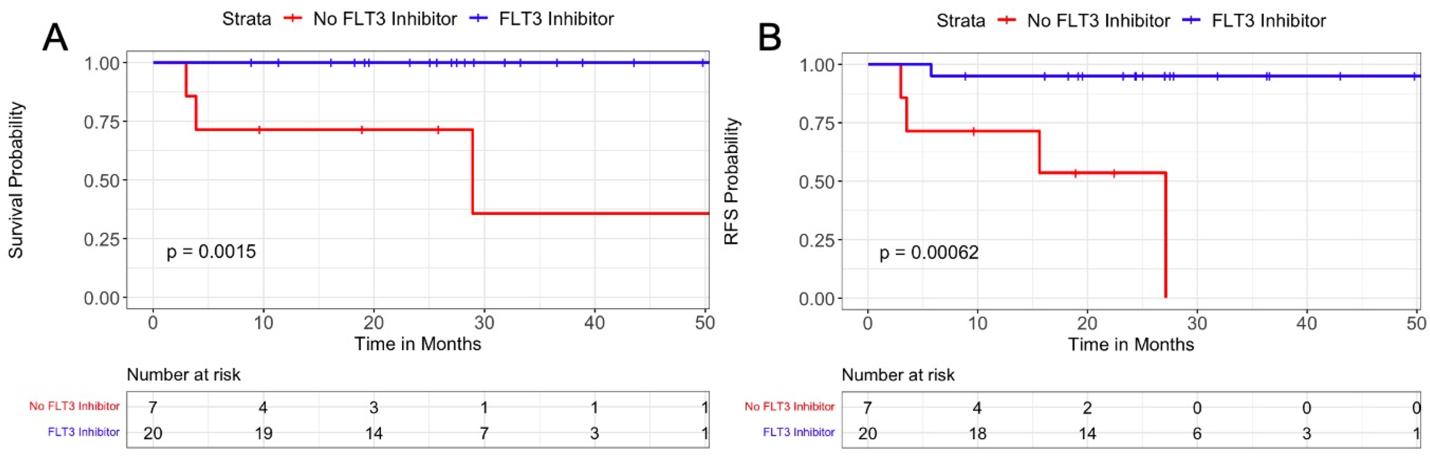


**Figure S3. Clinical impact of FLT3 inhibitor maintenance therapy after hematopoietic stem cell transplant in CR2 subgroup. (A) OS (B) RFS**

**
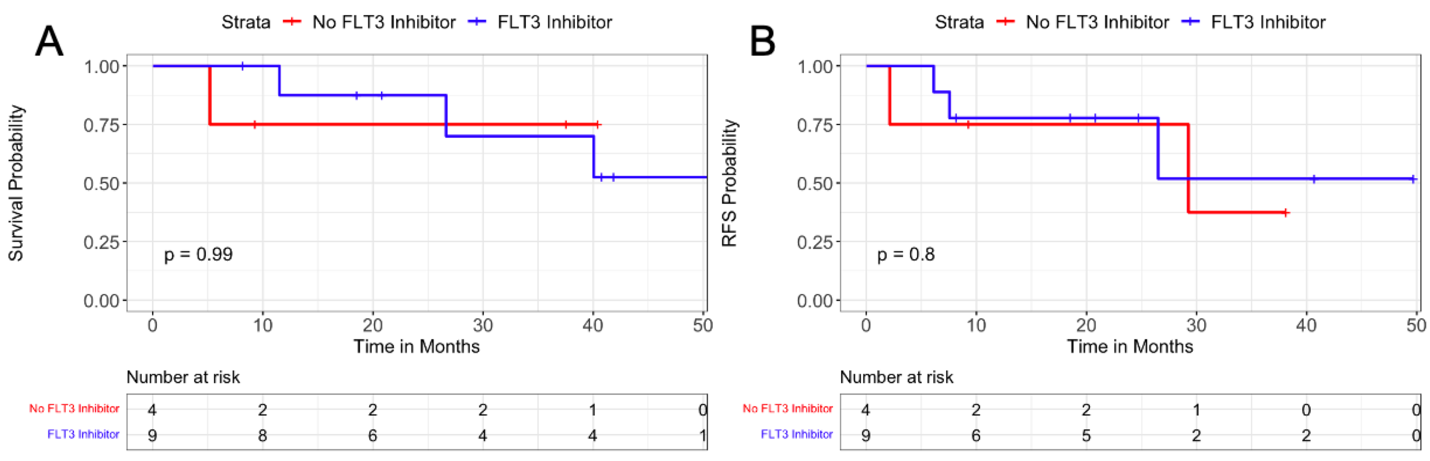
**

**Figure S4. Clinical impact of Gilteritinib maintenance therapy after hematopoietic stem cell transplant. (A) OS (B) RFS**


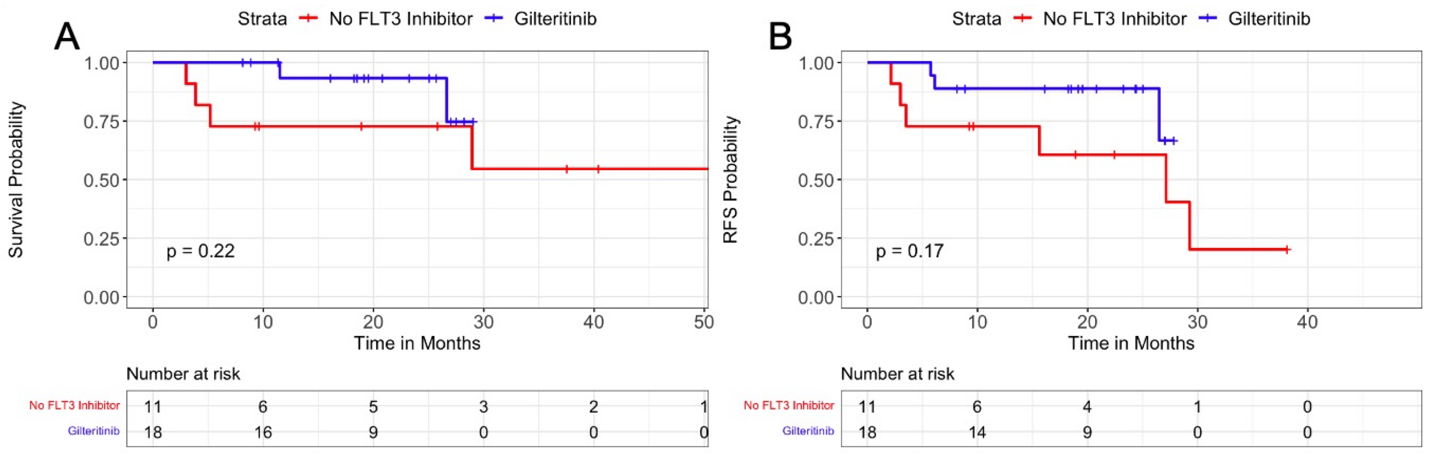


**Figure S5. KM Analysis of Overall Survival Predicted by FLT3 Inhibitor Use in Maintenance Therapy- USC FLT3 Inhibitor Use Group vs. Control OHSU Group**


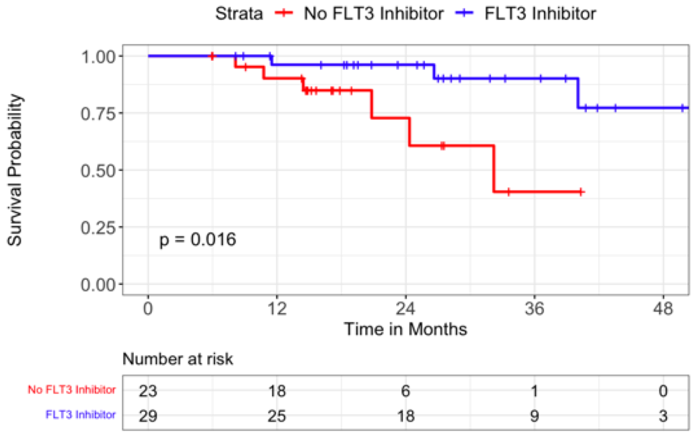


**Table S1. Baseline Characteristics USC FLT3 Inhibitor Group vs OHSU Group Without FLT3 Inhibitor**

| Comparative cohort :Demographics Stratified by FLT3 inhibitor use | | | | |
| --- | --- | --- | --- | --- |
|  | **No Post SCT FLT3(OHSU) (N=23)** | **Post SCT FLT3 used(USC) (N=29)** | **Total (N=52)** | **p value** |
| **Age** | 56.0 (24.0, 75.0) | 46.0 (30.0, 69.0) | 52.0 (24.0, 75.0) | 0.184^1^ |
| **Sex** |  |  |  | 0.780^2^ |
| Female | 12 (52.2%) | 17 (58.6%) | 29 (55.8%) |  |
| Male | 11 (47.8%) | 12 (41.4%) | 23 (44.2%) |  |
| **Race/Ethnicity** |  |  |  | < 0.001^3^ |
| Asian | 1 (4.3%) | 0 (0.0%) | 1 (1.9%) |  |
| Black | 0 (0.0%) | 4 (13.8%) | 4 (7.7%) |  |
| Hispanic | 1 (4.3%) | 14 (48.3%) | 15 (28.8%) |  |
| White | 21 (91.3%) | 11 (37.9%) | 32 (61.5%) |  |
| **Blast %** | 86.0 (51.0, 95.0) | 70.0 (10.0, 95.0) | 75.5 (10.0, 95.0) | 0.012^1^ |
| **FLT3 Mutation Type** |  |  |  | 1.000^2^ |
| ITD | 18 (78.3%) | 22 (75.9%) | 40 (76.9%) |  |
| TKD | 5 (21.7%) | 7 (24.1%) | 12 (23.1%) |  |
| **ELN Risk** |  |  |  | 0.467^3^ |
| Favorable | 3 (20.0%) | 5 (26.3%) | 8 (23.5%) |  |
| Intermediate | 4 (26.7%) | 8 (42.1%) | 12 (35.3%) |  |
| Adverse | 8 (53.3%) | 6 (31.6%) | 14 (41.2%) |  |
| **Median Follow-up** | 15.7 (6.0, 40.3) | 27.0 (8.2, 52.0) | 20.8 (6.0, 52.0) | 0.002^1^ |
| **Patient Status** |  |  |  | 0.161^2^ |
| Alive | 17 (73.9%) | 26 (89.7%) | 43 (82.7%) |  |
| Dead | 6 (26.1%) | 3 (10.3%) | 9 (17.3%) |  |
| **NPM1 Mutation** |  |  |  | 1.000^2^ |
| Negative | 12 (52.2%) | 15 (51.7%) | 27 (51.9%) |  |
| Positive | 11 (47.8%) | 14 (48.3%) | 25 (48.1%) |  |
| **Donor Type** |  |  |  | 0.003^2^ |
| Haplo SCT | 0 (0.0%) | 11 (37.9%) | 11 (21.2%) |  |
| MRD | 7 (30.4%) | 8 (27.6%) | 15 (28.8%) |  |
| MUD | 14 (60.9%) | 10 (34.5%) | 24 (46.2%) |  |
| MMUD | 2 (8.7%) | 0 (0.0%) | 2 (3.8%) |  |

1. Kruskal-Wallis rank sum test.

2. Fisher's Exact Test.
